# THE ROLE OF SUPPORT IN THE LIVED EXPERIENCE OF DEMENTIA CAREGIVERS: A SYSTEMATIC REVIEW AND THEMATIC SYNTHESIS

**DOI:** 10.1093/geroni/igac059.2100

**Published:** 2022-12-20

**Authors:** Paria Seyyedmirza, Clark Benson, Diane Ploch Emily, Anna Linden, Andrea Gilmore-Bykovskyi

**Affiliations:** University of Wisconsin-Madison, Madison, Wisconsin, United States; University of Wisconsin-Madison, Madison, Wisconsin, United States; University of Wisconsin-Madison, Madison, Wisconsin, United States; University of Wisconsin-Madison, Madison, Wisconsin, United States; University of Wisconsin-Madison, Madison, Wisconsin, United States

## Abstract

People living with dementia have complex care needs, which are primarily met by unpaid family caregivers. Family caregivers are often underprepared and under-supported in these roles and often experience negative health impacts associated with their caregiving responsibilities. Research suggests caregiving experiences and associated outcomes can be improved through the use of supportive resources which vary widely in design, access, and implementation. Yet how dementia caregivers perceive, identify, and experience supports in the context of their lived experience is less well understood. Understanding caregivers’ un-proscribed conceptualizations of “support” holds important implications for the optimal design of supportive interventions, which are often under-utilized. The objective of this qualitative evidence synthesis was to systematically identify, appraise, and synthesize evidence regarding dementia caregivers’ conceptualization of support through qualitative studies focused broadly on eliciting caregivers’ reports of lived experience. Forty-one qualitative studies were analyzed and synthesized according to methods suggested by Sandelowski (2007) and Graneheim & Lundman (2004). Six themes were identified and synthesized across included studies which include a range of domains from accessibility, awareness, usability, and match of informal and formal support for caregivers' needs and the needs of their care recipient. Caregivers conceptualized support broadly, extending beyond traditional resources to address aspects of their caregiving role. Findings demonstrate that caregivers readily distinguish between formal and informal support, but do not necessarily evaluate them uniformly and are perhaps focused on the fit of support that extends beyond the caregiving role and is more aligned with how caregivers view support in their daily lives.

